# Toxic and Essential Metals in Human Health and Disease 2021

**DOI:** 10.3390/biom12101375

**Published:** 2022-09-26

**Authors:** Jan O. Aaseth

**Affiliations:** 1 Department of Research, Innlandet Hospital Trust, P.O. Box 104, 2381 Brumunddal, Norway; jan.aaseth@inn.no; 2 Faculty of Health and Social Sciences, Inland Norway University of Applied Sciences, P.O. Box 400, 2418 Elverum, Norway

The Special Issue of *Biomolecules* called “Toxic and Essential Metals in Human Health and Disease 2021” represents a follow-up of the previous Special Issue with the name of “Toxic and Essential Metals in Human Health and Disease”. We intended to further explore the physicochemical and biological aspects of the elements, both in terms of the expanding appreciation of the essentiality of individual elements and the toxicity of excessive amounts of each element. The nutritional importance and metabolic complexities of the trace and mineral elements are reflected by the diversity of conditions recognized as being affected by derangements or deficiencies of these substances, such as neurodegenerative diseases, dermatological conditions, carcinogenic processes, osteoporosis, cardiovascular diseases, and endocrine dysfunctions. The ability of some trace elements to interfere with the biochemical and physiological utilization of molecular oxygen continues to sustain considerable research interest.

During the past decades, we have witnessed remarkable advances in the field of trace element research. Advances in analytical techniques have made it easier to measure concentrations of essential and of non-essential elements with great accuracy in biological fluids and samples. Among the current challenges today are the insufficient nutritional availability of iron, zinc and selenium in significant segments of the human population. At the same time, increasing urbanization poses an increased burden to population groups regarding some toxic elements, such as cadmium and mercury. Furthermore, among the intriguing but still unanswered questions are the molecular mechanisms underlying the apparent cardioprotective and anticarcinogenic properties of selenium.

In the present Special Issue, sixteen contributions were published, including six reviews and ten original research articles spanning an extensive series of essential and non-essential metals.

In their research article that was selected as Editor’s Choice, Adamson et al. [1] present an analysis of the impact of Cu dyshomeostasis on olfactory function, neurogenesis, and neurochemical balance. They used adult rats with copper deficiency or copper overload as animal models. Some interesting observations were that copper deficiency increased neural proliferation in the subventricular zone and created newly differentiated neurons in the olfactory bulb, whereas copper overload caused opposite alterations. The authors reason that the observed altered neurogenesis could contribute to olfactory dysfunction.

Gigliuto et al. [2] studied, in NaCl-solutions in vitro, the interactions of dopamine as a ligand (L) with various metal compounds including CH_3_Hg^+^, Mg^2+^, Ca^2+^, and Sn^2+^. For the compound CH_3_Hg^+^, their speciation model identified the formation of the ternary complex CH_3_Hg-L-Cl. Upon further evaluation of the sequestering ability of dopamine towards the investigated cations, they disclosed the following order of affinity: Sn^2+^ > CH_3_Hg^+^ > Ca^2+^ > Mg^2+^. Their observations imply relevance also for in vivo effects of dopamine.

Alexandra Kuzan and coworkers [3] present analyses of macro- and microelements in aorta sections of people who died a sudden death from cardiovascular diseases. It was observed that with the increasing age of the patients, the calcium content of the artery increased, while the content of copper and iron decreased. High correlations were observed for some pairs of parameters, especially in women, typically seen for Fe–Cu, and Ca–Cd. The degree of atherosclerosis correlated negatively with magnesium. Selenium was not quantified in their studies.

In a review, Alehagen et al. [4] discuss the role of adequate intake of selenium in the prevention of cardiovascular disease, cancer, and in the maintenance of optimal immune functions. Decreased activity of selenoenzymes, leading to imbalance between antioxidative defense and reactive oxygen species, might be associated with a mild to moderate chronic inflammation together with the accelerated development of cellular senescence and age-related diseases. The reduced cardiovascular mortality ten years after supplementation with selenium for four years that they previously observed [5] might be related to the antioxidative effects of selenoenzymes and the proposed role in the preservation of telomere length.

Tania C. Araujo-Jorge et al. [6] present a compilation of studies in relation to the cardiomyopathy characterizing chronic Chagas disease caused by *Trypanosoma cruzi* infection. Experimental and clinical data indicate that selenium may be used as a complementary therapy to prevent heart failure. The pathophysiology of Chagas cardiomyopathy appears to involve a complex immunological interplay involving the multifunctional cytokine TGF-β, the latter mediator operating as regulator of the immune response and the precipitated fibrosis. Selenoproteins induced by selenium supplementation could act as antioxidants to regulate the increased reactive oxygen stress present in the inflammation accompanying Chagas cardiomyopathy.

Tinkov et al. [7] present blood serum and urinary levels of trace elements in normal-weight and obese women in relation to metabolic risk factors. The obese subjects were characterized by insulin resistance and increased blood pressure and triglyceride concentrations, while their serum iron, magnesium, selenium and zinc levels were lower than the levels in lean controls. The authors suggest that the disturbances in trace element and mineral status, at least in part, could contribute to the metabolic risks in the obese subjects.

Bjørklund et al. [8] review the frequency of iron deficiency in obesity and after bariatric surgery. Obese subjects often have a low-grade inflammation accompanied by increased levels of the acute-phase reactant hepcidin which can induce reduced iron absorption. Obesity surgery with gastric bypass or sleeve gastrectomy can induce malabsorption and may accentuate an iron deficiency. After bariatric surgery, the iron status should be regularly monitored, and the treated subjects should be motivated to use adequate iron supplementation.

Sigrun Henjum and coworkers [9] have studied iron status in vegetarians, vegans and pescatarians in Norway, by determining several biomarkers in blood serum (ferritin, serum-iron, total iron binding capacity, and transferrin saturation). Their conclusion is that although the participants were eating a plant-based diet, the majority had sufficient iron status. Nonetheless, female vegans and vegetarians of reproductive age should have their iron status regularly monitored.

Yasuda et al. [10] present analytical research on the toxic metal burden in infants/children and the relationship of the determined levels to those in their mothers for 77 child/mother pairs. As for mercury, mean concentration in infants/children was comparable to that in their mothers. However, the burden levels of lead, cadmium and aluminum in infants/children were approximately three times higher than those in their mothers. In contrast, some essential metal levels such as zinc, magnesium and calcium in infants/children were lower than those in their mothers. The toxic metal burdens represent serious concerns for the neurodevelopment of the children, and larger epidemiological and intervention studies are requested.

Nordberg and Nordberg [11] review the toxicology of cadmium, underlining the need for preventive measures in view of the growing risk of metal exposure during the ongoing global climate change. These authors already contributed evidence in the 1970s concerning the cadmium binding to the protein metallothionein and the metal-induced synthesis of this protein in tissues. The binding of cadmium to metallothionein in tissues prevents some toxic effects, but metallothionein can increase the transport of cadmium to the kidneys. However, later studies have shown the importance of the Cd/Zn ratio in MT for expression of Cd toxicity in the kidneys. Thus, a study of the impact of zinc status on the risk of kidney dysfunction in a cadmium-exposed cohort disclosed that the risks were low when the zinc status was good but high when the zinc status was poor. This review summarizes current evidence in risk assessment and calls for improved preventive measures against the adverse effects of cadmium exposures in humans and animals. 

Aaseth et al. [12], in a review, underscore the insufficient knowledge regarding the response of the aged kidney to environmental toxicants such as cadmium, mercury, and lead, and present proposed mechanisms of the aged kidney to metal pollutants. Human exposure to these toxicants is practically unavoidable in our industrialized society, and it may be hypothesized that exposure of individuals with reduced GFR will result in additional reductions in renal function. Available data appear to show an association between exposure to cadmium, mercury, and/or lead and an increase in incidence and severity of renal disease in elderly individuals. Furthermore, some physiological thiols, as well as adequate selenium status, appear to exert a protective action. Further studies providing improved insight into the mechanisms by which nephrotoxic metals are handled by aging kidneys, as well as possibilities for therapeutic protection, are of utmost importance.

In recent years, the mechanisms of Mn-induced neurotoxicity have been extensively studied by the research group of Michael Aschner [13]. The neurotoxicity of Mn involves i.a. neuroinflammation, oxidative and endoplasmic reticulum stress, and mitochondrial dysfunction. Existing data on the impact of Mn exposure on gut microbiota biodiversity, bacterial metabolite production, and gut wall permeability are discussed in their review [13]. A Mn-induced increase in Bacteroidetes and a reduced Firmicutes/Bacteroidetes ratio may increase lipopolysaccharide levels. Moreover, in addition to increased systemic lipopolysaccharide levels, Mn is capable of potentiating lipopolysaccharide neurotoxicity. Furthermore, a recent study demonstrated that healthy microbiome transplantation alleviates Mn-induced neurotoxicity. Existing data may lead to the hypothesis that gut microbiota represent a potential target of Mn toxicity, although more detailed studies are required to characterize the interplay between Mn exposure and the gut.

In a research article, Viviana Poliseno et al. [14] present the synthesis of derivatives of tenuazonic acid, especially tenuazonic-donepezil derivatives. They are about to evaluate these compounds as regards their possible protective potential against Alzheimer’s disease. These novel compounds exert biological activity in the micromolar range and appear to be orally absorbed. One of the compounds has been further investigated as a chelating agent towards copper (II), zinc (II) and iron (III) and have shown good chelating ability. The tenuazonic acid motif can be considered an interesting building block in the search for innovative multi-functional anti-neurodegenerative drugs.

Bosiacki et al. [15] present studies on aging rats subjected to physical training in cold water 5 ± 2 °C and in water with thermal comfort temperature (36 ± 2 °C). Control animals were kept in a sedentary condition. The effect of cold water immersion appeared to be gender dependent. In females, it decreased Ca and Mg content in bones while increasing 17-β estradiol and 1,25-dihydroxyvitamin D_3_ levels. In males, cold water swimming decreased PTH and resulted in a decrease in phosphorus content in bones, and an increase in 1,25-dihydroxyvitamin D_3_, and it may have positive consequences for the prevention of elderly sarcopenia.

Condeles and Toledo Junior [16] present their observation that the labile iron pool can react with peroxynitrite, thereby leading to decreased yield of peroxynitrite-derived oxidants. In their follow-up study, they found that the reaction between the labile iron pool and peroxynitrite has catalytic characteristics, and they estimated the rate constant of the reaction to be in the range of 10^6^ to 10^7^ M^−1^s^−1^. Together, these observations suggest that the labile iron pool represents a constitutive peroxynitrite reductase system, as observed in their model of murine macrophage cells (RAW 264.7).

Belotti et al. [17] report studies on a zinc transporter (Zrt2) of the ZIP family, this transporter being essential for *Candida albicans*. The transporter is localized in the plasma membrane and contains eight transmembrane domains corresponding to the amino acid sequence 126–215. This amino acid sequence is considered to contain three metal binding motifs. Model peptides were studied to elucidate coordination properties of their Zn^2+^ and Cu^2+^ complexes, with the aim of identifying the most effective metal binding site among the three fragments. In the native Zrt2 protein, the Ac-GPHTHSHFGD-NH_2_ region of the Zrt2 loop had the highest metal binding affinity, demonstrating that three alternating histidines separated by only one residue bind Zn^2+^ and Cu^2+^ more strongly than the region in which three histidines are separated by two and three residues. All studied Zrt2 loop fragments have lower affinity towards Zn^2+^ than the zinc(II) binding site on the Zrt1 transporter. In addition, all three Zrt2 regions bind Zn^2+^ and Cu^2+^ with comparable affinity below pH 5 and, therefore, may equally contribute to the metal acquisition under the most acidic conditions in which the Zrt2 transporter is expressed.

## Data Availability

Not applicable.
